# Supplementary material for: Mosquito immune cells enhance dengue and Zika virus infection in Aedes aegypti
Source: Nat Commun. 2025 Jul 1;16:5891. doi: 10.1038/s41467-025-61139-9 (PMC12214649; doi:10.1038/s41467-025-61139-9)
Supplement: Supplementary file 1 — Supplementary Information [file 41467_2025_61139_MOESM1_ESM.pdf]

Supporting Information

Mosquito immune cells enhance dengue and Zika virus infection in *Aedes aegypti*

David R. Hall[1,2,#], Rebecca M. Johnson[3,#], Hyeogsun Kwon[2,#], Zannatul Ferdous[3], S. Viridiana Laredo-Tiscareño[4], Bradley J. Blitvich[4], Doug E. Brackney[3], Ryan C. Smith[2,*]

[1]Interdepartmental Program in Genetics and Genomics, Iowa State University, Ames, Iowa
[2]Department of Plant Pathology, Entomology and Microbiology, Iowa State University, Ames, Iowa
[3]Center for Vector-Borne and Zoonotic Diseases, Department of Entomology, The Connecticut Agricultural Experiment Station, New Haven, Connecticut
[4]Department of Veterinary Microbiology and Preventative Medicine, Iowa State University, Ames, Iowa

[#]These authors contributed equally and are listed alphabetically

*Corresponding author: smithr@iastate.edu

Included supporting information

Supplemental Figures

Figure S1. Stable depletion of granulocytes by clodronate liposome injection.

Figure S2. Individual experiments examining the effects of phagocyte depletion on DENV and ZIKV midgut infection.

Figure S3. Influence of phagocyte depletion on mosquito survival following blood-feeding and infection.

Figure S4. Phagocytic granulocytes associate the with the salivary glands and ovaries of DENV-infected mosquitoes.

Figure S5. Attachment of DiI-labeled hemocytes to mosquito tissues.

Figure S6. Evaluation of virus infection to mosquito tissues using FFAs.

Figure S7. Evaluation of virus infection of mosquito tissues after cell transfer using FFAs.

Supplemental Tables

Table S1. Summary of blood meal virus titers for infection experiments.

Table S2. Primers used to assess virus titers in midgut and transfer experiments.

Table S3. Primers used to assess virus titers in infection experiments.

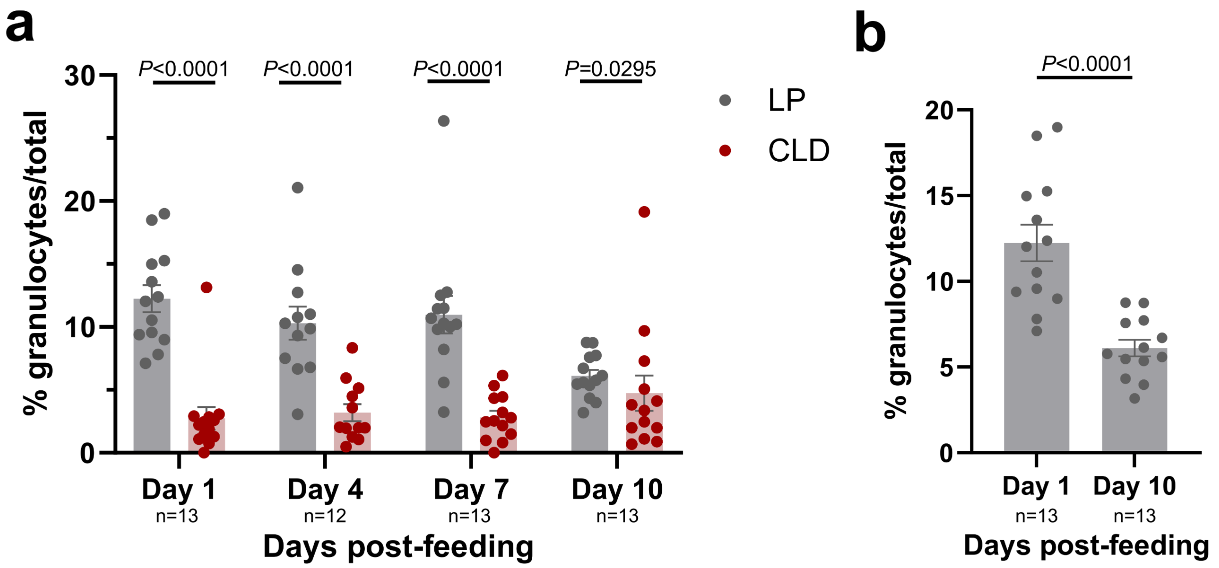

Figure S1. Stable depletion of granulocytes by clodronate liposome injection. To determine the duration and efficacy of granulocyte depletion by clodronate liposomes in blood-fed *Ae. aegypti*, mosquitoes were first injected with either clodronate liposomes (CLD) to deplete phagocytic granulocyte populations or control liposomes (LP), then blood-fed at 24 hours post-injection. Hemolymph was perfused at days 1, 4, 7, and 10 post-feeding and the percentage of granulocytes of the total hemocyte population from individual mosquitoes (n) were determined using a hemocytometer (**a**). The percentage of granulocytes were analyzed using a Multiple Mann-Whitney test with a two-stage step-up (Benjamini, Krieger, and Yekutieli) to correct for multiple comparisons. Exact *P* values are displayed in the figure. (**b**) Additional analysis of the percentage of granulocytes in individual control LP-injected mosquitoes (n) demonstrates that the percentage of granulocytes was significantly lower between day 1 and day 10, suggesting that there is a decline in the percentage of granulocytes with age. Data were analyzed using a two-tailed Mann-Whitney test. Exact *P* values are displayed in the figure. For both **a** and **b**, individual mosquitoes are displayed as dots, with the bar graphs displaying the mean ±SEM of two independent experiments. Source data are provided as a Source Data file.

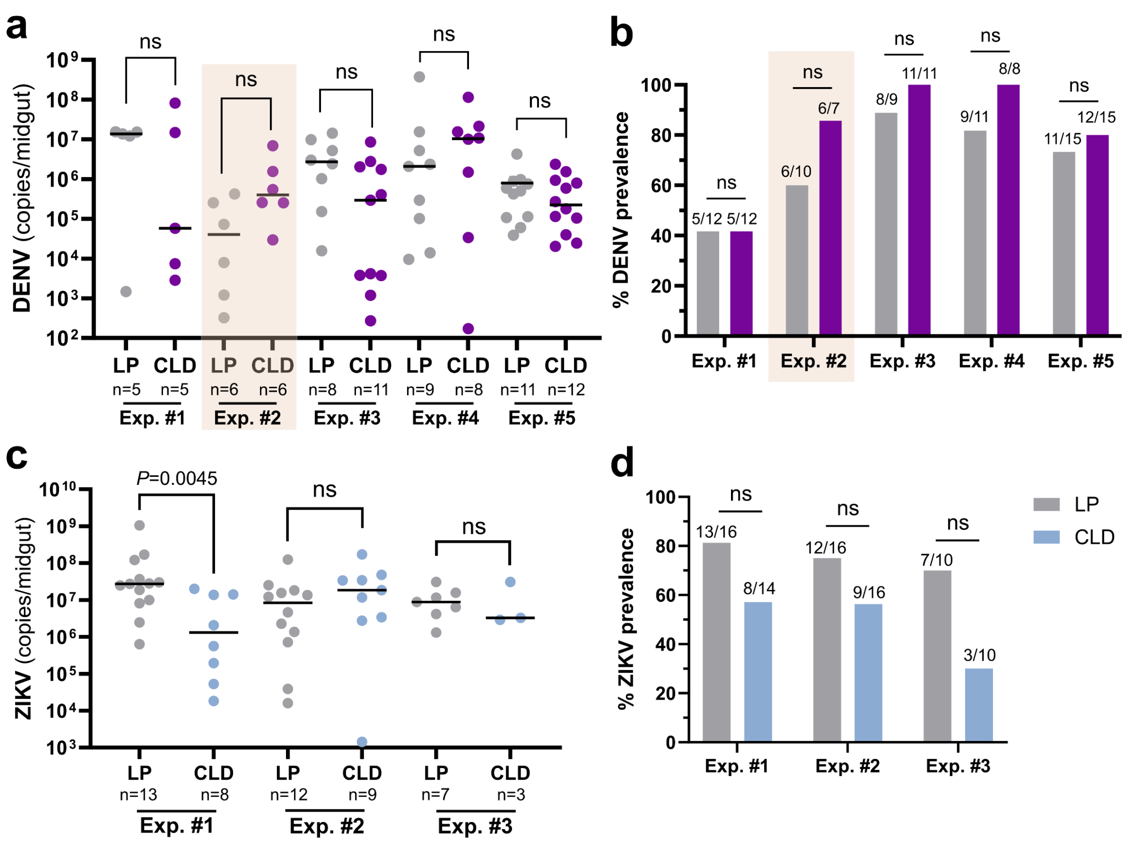

Figure S2. Individual experiments examining the effects of phagocyte depletion on DENV and ZIKV midgut infection. A total of five independent infection experiments were performed to examine the effects of phagocyte depletion on DENV midgut infection. For each individual experiment, DENV copy numbers (**a**) and infection prevalence (**b**) are displayed for individual mosquitoes injected with either control liposomes (LP) or clodronate liposomes (CLD). Similarly, ZIKV copy numbers (**c**) and infection prevalence (**d**) are displayed for LP and CLD backgrounds from three independent experiments. For data **a** and **c**, dots represent individual midgut samples with the median denoted by the black line. Viral copy numbers in individual experiments were analyzed using a two-tailed Mann-Whitney test. Exact *P* values are displayed in the figure where applicable; ns, not significant. To compare between experiments, LP samples from each experiment were analyzed using Kruskal-Wallis with a Dunn's post-test, identifying that Exp. #2 (shaded orange) was statistically different than the other DENV experiments and was excluded from the pooled data included in **Fig. 1.** For **b** and **d**, infection prevalence data from individual experiments were examined using a two-sided Fisher's exact test. ns, not

significant. Source data and statistical comparisons between replicates are provided as a Source Data file.

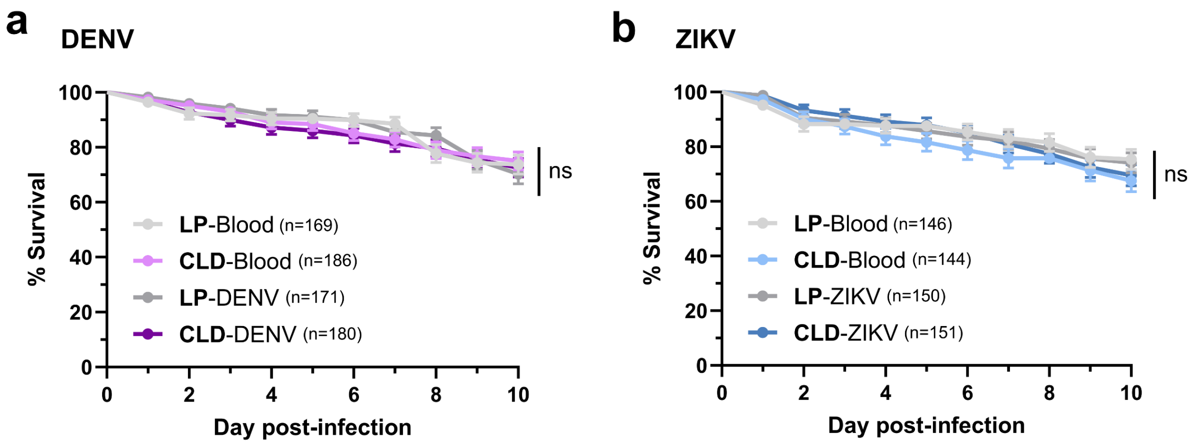

Figure S3. Influence of phagocyte depletion on mosquito survival following blood-feeding and infection. Adult mosquitoes were treated with either LP or CLD, then challenged with DENV (**a**) or ZIKV (**b**) and compared to mosquitoes fed on non-infected blood. Survival was monitored in individual mosquitoes every day for 10 days. No differences in mosquito mortality were observed among treatments (**a** and **b**), indicating that phagocyte depletion did not impact mosquito survival following viral infection. Error bars represent the mean ± SEM of three independent replicates, with approximately 50 female mosquitoes were used for each replicate. n= number of individual mosquitoes examined for each experimental condition. Survival data were analyzed using a log-rank (Mantel-Cox) test. ns, not significant. Source data are provided as a Source Data file.

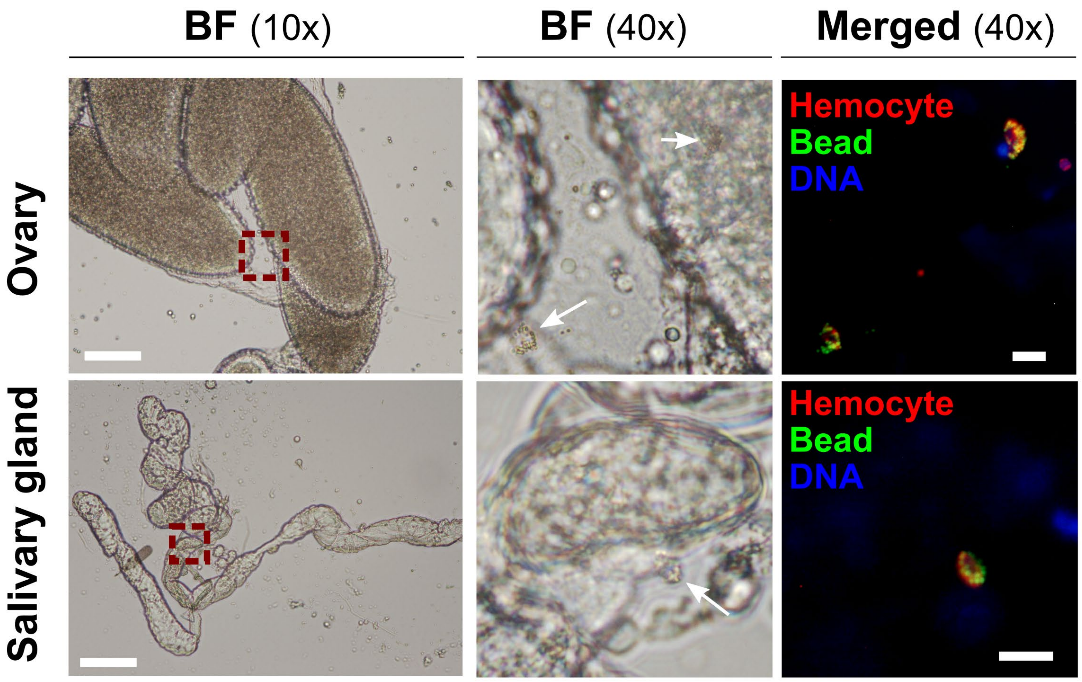

Figure S4. Phagocytic granulocytes associate the with the salivary glands and ovaries of DENV-infected mosquitoes. To examine hemocyte attachment to mosquito tissues, ZIKV-infected mosquitoes were injected with CM-DiI (red) and fluorescent beads (green) at 7 days post-infection to identify phagocytic granulocyte populations. Following staining *in vivo*, ovary and salivary gland tissues were dissected to examine granulocyte attachment to each respective tissue and mounted using ProLong®Diamond Antifade mountant with DAPI (blue). Red dashed line boxes denote the field of view at 40x magnification, with white arrows used to indicate attached phagocytic hemocytes in the bright field (BF) image where applicable. Scale bars denote 100 µm for 10x images and 10 µm for 40x images.

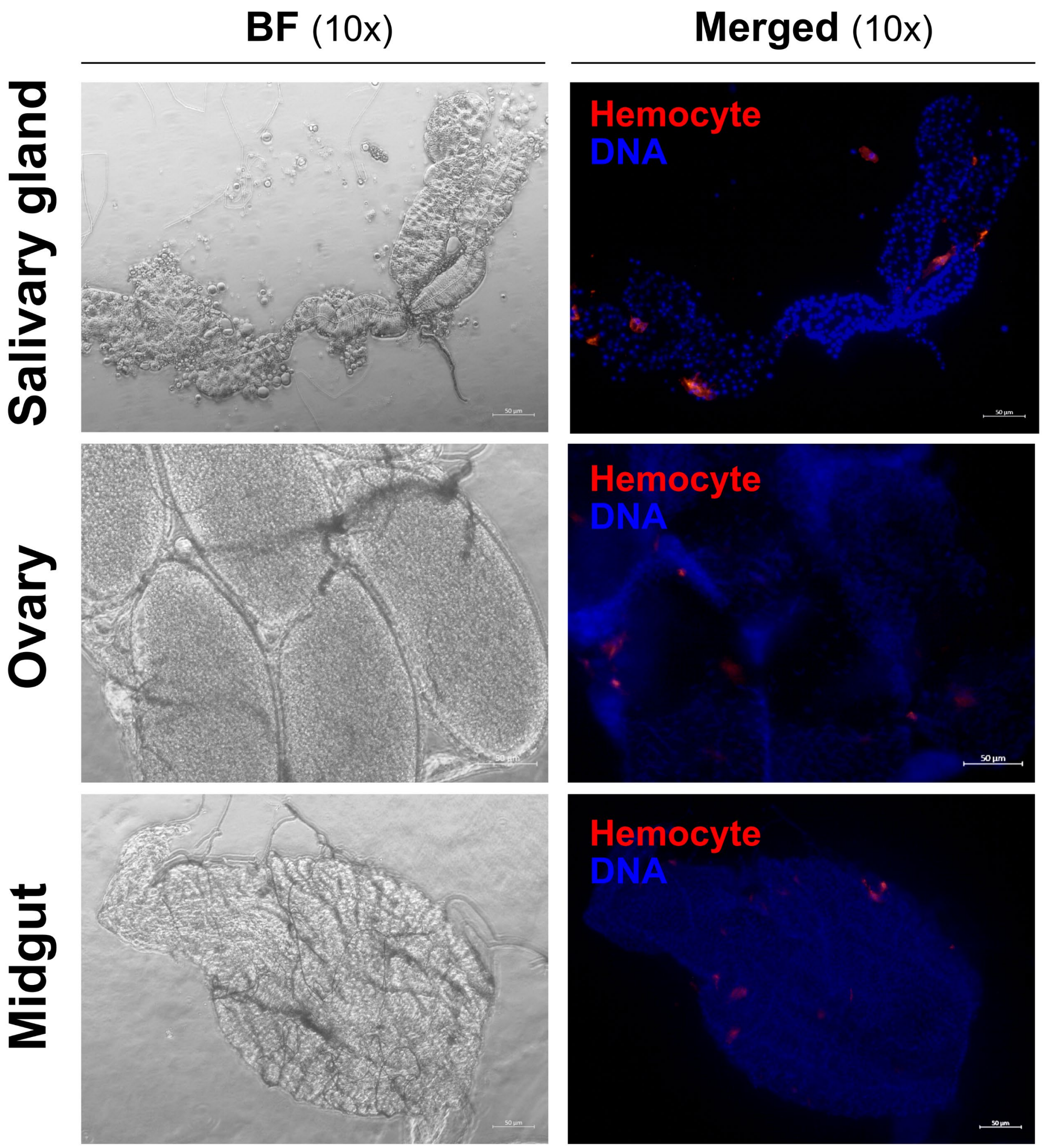

Figure S5. Attachment of CM-DiI-labeled hemocytes to mosquito tissues. Hemocyte attachment to the salivary glands, ovary, and midgut was evaluated by staining hemocytes with CM-DiI (red) and nuclei with Hoechst 33342 (blue). Attached hemocytes were quantified and presented in **Fig. 3c**.

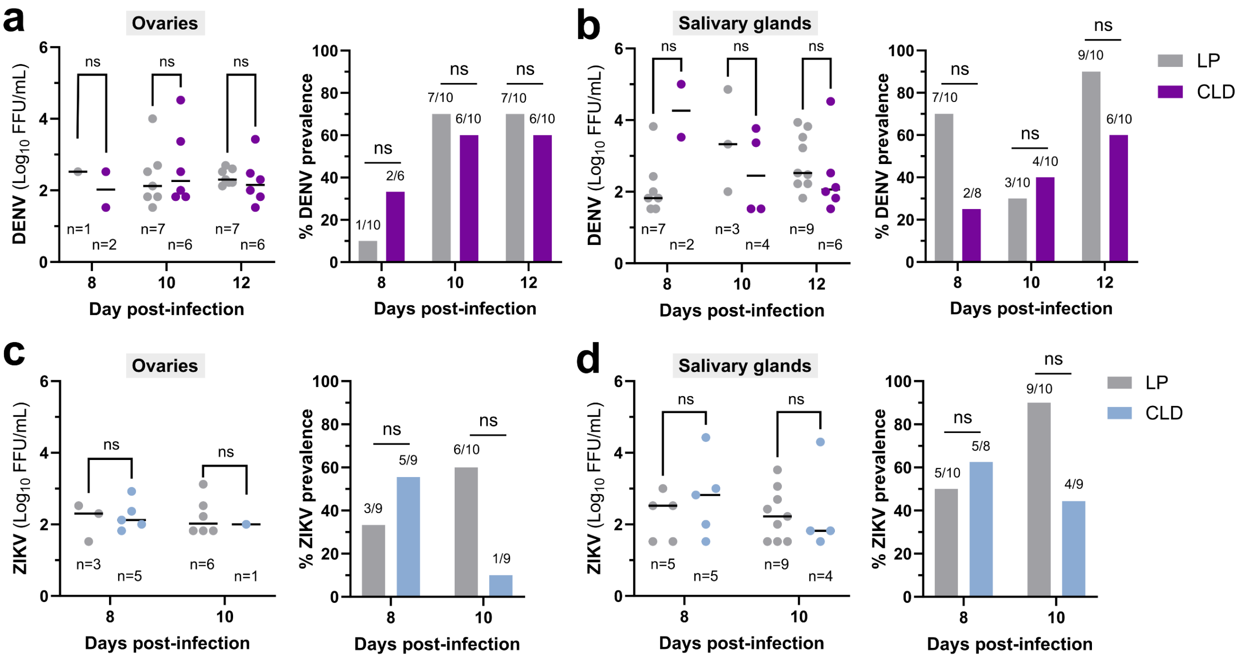

Figure S6. Evaluation of virus infection to mosquito tissues using FFAs. After oral challenge with DENV or ZIKV, mosquitoes were injected with control (LP) or clodronate liposomes (CLD) at 3 days post-infection. Virus dissemination was examined in a subset of samples for the ovaries (**a** and **c**) and salivary glands (**b** and **d**) at 8, 10, and 12 days post-infection for DENV (**a** and **b**), or 8- and 10-days post-infection for ZIKV (**c** and **d**). For each subpanel, viral titers are examined via focus-forming assays (FFAs), with each dot representing the titer of each individual mosquito samples (n) and the median marked by the black line. Infection prevalence is displayed in bar graphs with the number of infected tissues of the total analyzed depicted above each bar. Viral titers were analyzed using Multiple Mann-Whitney tests with a two-stage step-up (Benjamini, Krieger, and Yekutieli) to correct for multiple comparisons. Infection prevalence data were analyzed using a two-sided Fisher's exact test. ns, not significant. Source data are provided as a Source Data file.

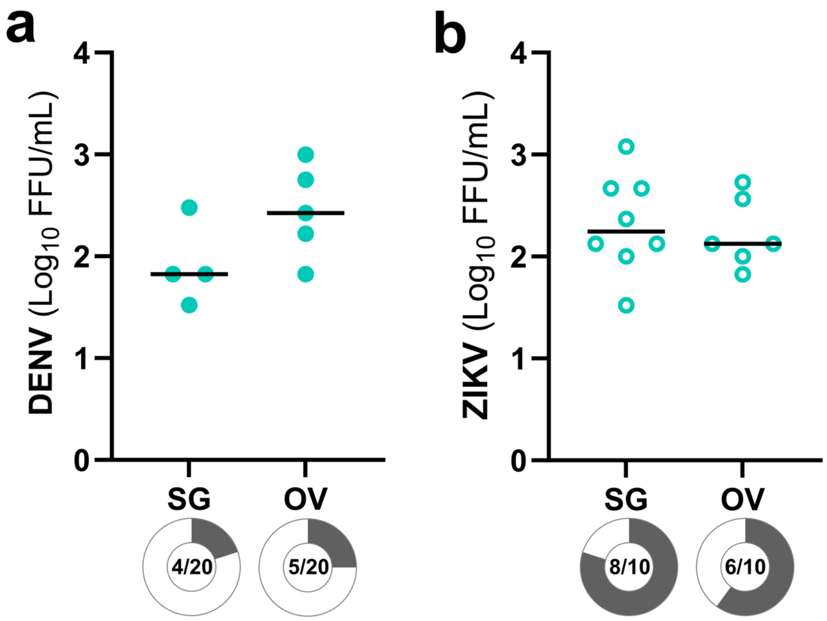

Figure S7. Evaluation of virus infection of mosquito tissues after cell transfer using FFAs. Following the transfer of the CELL fraction from infected mosquitoes to naïve mosquitoes, DENV (**a**) or ZIKV (**b**) titers were examined in salivary gland (SG) or ovary tissues (OV) using focus-forming assays (FFAs) at four days post-transfer. Each circle corresponds to the titer of each individual mosquito sample (n), with the median marked by the black line. Shaded areas of circles under each experimental condition display the infection prevalence, with the number infected of the total number of mosquitoes examined displayed for each treatment. Source data are provided as a Source Data file.

Table S1. Summary of blood meal virus titers for infection experiments.

Experiment	Virus	Replicate	GC/ml	FFU/ml
CLD Midgut	DENV	1	7.8×10^{9}	-
CLD Midgut	DENV	2	7.7×10^{9}	-
CLD Midgut	DENV	3	-	-
CLD Midgut	DENV	4	-	-
CLD Midgut	DENV	5	6.19×10^{8}	-
CLD Midgut	ZIKV	1	1.7×10^{9}	-
CLD Midgut	ZIKV	2	7.1×10^{9}	-
CLD Midgut	ZIKV	3	9.9×10^{9}	-
CLD dissemination	DENV	1	-	3.0×10^{7}
CLD dissemination	DENV	2	-	3.7×10^{8}
CLD dissemination	DENV	3	-	5.0×10^{8}
CLD dissemination	ZIKV	1	-	1.3×10^{8}
CLD dissemination	ZIKV	2	-	1.7×10^{8}

GC/ml = gene copies per ml

FFU/ml = focus forming units per ml

- = not determined

Table S2. Primers used to assess virus copy numbers in midgut and transfer experiments.

Primer	Sequence
DENV-2 F	5'-GCATATTGACGCTGGGARAGAC-3'
DENV-2 R	5'-TTCTGTGCCTGGAATGATGCTG-3'
DENV-2 Probe	5'-[6FAM]CAGAGATCCTGCTGTC[BHQ1]-3'
ZIKV F	5'-TTGTCATGATACTGCTGATTGC-3'
ZIKV R	5'-CCTTCCACAAAGTCCCTATTGC-3'
ZIKV Probe	5'-[6FAM]CGGCATACAGCATCAGGTGCATAGGAG[BHQ1]-3'

Table S3. Primers used to assess virus copy numbers in infection experiments.

Primer	Sequence
DENV-2 F	5’-CATGGCCCTKGTGGCG-3’
DENV-2 R	5’-CCCCATCTYTTCAGTATCCCTG-3’
DENV-2 Probe	5’-[FAM] TCCTTCGTTTCCTAACAATCC [BHQ1]-3’
ZIKV 1087 F	5’-CCGCTGCCCAACACAAG-3’
ZIKV 1163c R	5’-CCACTAACGTTCTTTTGCAGACAT-3’
ZIKV 1108 Probe	5’-[FAM] AGCCTACCTTGACAAGCAGTCAGACACTCAA [BHQ1]-3’
