# Supplementary material for: Mosquito immune cells enhance dengue and Zika virus infection in Aedes aegypti
Source: Nat Commun. 2025 Jul 1;16:5891. doi: 10.1038/s41467-025-61139-9 (PMC12214649; doi:10.1038/s41467-025-61139-9)
Supplement: Supplementary file 2 — Reporting summary [file 41467_2025_61139_MOESM2_ESM.pdf]

nature portfolio

Corresponding author(s): Smith, Ryan C.

Last updated by author(s): Jun 2, 2025

Reporting Summary

Nature Portfolio wishes to improve the reproducibility of the work that we publish. This form provides structure for consistency and transparency in reporting. For further information on Nature Portfolio policies, see our Editorial Policies and the Editorial Policy Checklist.

Statistics

For all statistical analyses, confirm that the following items are present in the figure legend, table legend, main text, or Methods section.

n/a	Confirmed	
☐	☒	The exact sample size (*n*) for each experimental group/condition, given as a discrete number and unit of measurement
☐	☒	A statement on whether measurements were taken from distinct samples or whether the same sample was measured repeatedly
☐	☒	The statistical test(s) used AND whether they are one- or two-sided<br>*Only common tests should be described solely by name; describe more complex techniques in the Methods section.*
☐	☒	A description of all covariates tested
☐	☒	A description of any assumptions or corrections, such as tests of normality and adjustment for multiple comparisons
☐	☒	A full description of the statistical parameters including central tendency (e.g. means) or other basic estimates (e.g. regression coefficient) AND variation (e.g. standard deviation) or associated estimates of uncertainty (e.g. confidence intervals)
☐	☒	For null hypothesis testing, the test statistic (e.g. *F*, *t*, *r*) with confidence intervals, effect sizes, degrees of freedom and *P* value noted<br>*Give P values as exact values whenever suitable.*
☒	☐	For Bayesian analysis, information on the choice of priors and Markov chain Monte Carlo settings
☒	☐	For hierarchical and complex designs, identification of the appropriate level for tests and full reporting of outcomes
☒	☐	Estimates of effect sizes (e.g. Cohen's *d*, Pearson's *r*), indicating how they were calculated

Our web collection on statistics for biologists contains articles on many of the points above.

Software and code

Policy information about availability of computer code

Data collection	*Provide a description of all commercial, open source and custom code used to collect the data in this study, specifying the version used OR state that no software was used.*
Data analysis	All data visualization and statistical analysis was performed using GraphPad Prism v10.4.1

For manuscripts utilizing custom algorithms or software that are central to the research but not yet described in published literature, software must be made available to editors and reviewers. We strongly encourage code deposition in a community repository (e.g. GitHub). See the Nature Portfolio guidelines for submitting code & software for further information.

Data

Policy information about availability of data

All manuscripts must include a data availability statement. This statement should provide the following information, where applicable:

- Accession codes, unique identifiers, or web links for publicly available datasets
- A description of any restrictions on data availability
- For clinical datasets or third party data, please ensure that the statement adheres to our policy

All data supporting the findings of this study are available as Source Data that are included within the paper and its Supplementary Information.

Research involving human participants, their data, or biological material

Policy information about studies with human participants or human data. See also policy information about sex, gender (identity/presentation), and sexual orientation and race, ethnicity and racism.

Reporting on sex and gender	N/A
Reporting on race, ethnicity, or other socially relevant groupings	N/A
Population characteristics	N/A
Recruitment	N/A
Ethics oversight	N/A

Note that full information on the approval of the study protocol must also be provided in the manuscript.

Field-specific reporting

Please select the one below that is the best fit for your research. If you are not sure, read the appropriate sections before making your selection.

☒ Life sciences ☐ Behavioural & social sciences ☐ Ecological, evolutionary & environmental sciences

For a reference copy of the document with all sections, see nature.com/documents/nr-reporting-summary-flat.pdf

Life sciences study design

All studies must disclose on these points even when the disclosure is negative.

Sample size	Sample sizes (defined as the number of individual mosquitoes examined) are depicted for each experiment and are based on experimental numbers from previously published work.
Data exclusions	No data were excluded from our analysis.
Replication	All experiments were performed with at least 2 independent biological replicates. Midgut infection experiments were performed with 3+ independent biological experiments.
Randomization	Mosquito infection experiments were conducted in which mosquitoes were fed at random, then sorted to collect those that were fed from those that were non-fed. Surviving mosquitoes were analyzed at their respective time points.
Blinding	Due to limited personnel performing experiments, blinding was not possible.

Reporting for specific materials, systems and methods

We require information from authors about some types of materials, experimental systems and methods used in many studies. Here, indicate whether each material, system or method listed is relevant to your study. If you are not sure if a list item applies to your research, read the appropriate section before selecting a response.

Materials & experimental systems

n/a	Involved in the study	
☐	☒	Antibodies
☐	☒	Eukaryotic cell lines
☒	☐	Palaeontology and archaeology
☐	☒	Animals and other organisms
☒	☐	Clinical data
☒	☐	Dual use research of concern
☒	☐	Plants

Methods

n/a	Involved in the study	
☒	☐	ChIP-seq
☒	☐	Flow cytometry
☒	☐	MRI-based neuroimaging

Antibodies

Antibodies used	Immunofluorescence Assays (IFAs)<br>-mouse anti-DENV monoclonal antibody (clone 3H5-1; BEI Resources)

	-goat anti-mouse IgG (H+L) Alexa Fluor 568 secondary antibody (Thermo Fisher)<br><br>Focus Forming Assays (FFAs)<br>-mouse anti-flavivirus group antibody (D1-4G2-4-15; Millipore Sigma MAB10216, NovusBio NBP2-52709)<br>-goat anti-mouse IgG (H+L) Alexa Fluor 488 secondary antibody (Thermo Fisher)<br>-goat anti-mouse IgG (H+L) Alexa Fluor 568 secondary antibody (Thermo Fisher)
Validation	All antibody reagents were validated by their commercial supplier, with validation information on the supplier websites.

Eukaryotic cell lines

Policy information about cell lines and Sex and Gender in Research

Cell line source(s)	Vero cells (African green monkey kidney cells)<br>C6/36 cells (Aedes albopictus)
Authentication	Authentication was performed by the supplier.
Mycoplasma contamination	The cell lines were originally tested by the supplier and have no mycoplasma contamination.
Commonly misidentified lines<br>(See ICLAC register)	No commonly misidentified cell lines were used in this study.

Animals and other research organisms

Policy information about studies involving animals; ARRIVE guidelines recommended for reporting animal research, and Sex and Gender in Research

Laboratory animals	All experiments were performed using adult female Aedes aegypti (Orlando strain) mosquitoes. No vertebrate animals were used in our study.
Wild animals	N/A
Reporting on sex	All experiments were performed using adult female mosquitoes. Only female mosquitoes can blood-feed, making them susceptible to oral infections with arbovirus, such as dengue (DENV) and Zika (ZIKV) virus, which were the focus of our study.
Field-collected samples	N/A
Ethics oversight	All experiments were approved by the Institutional Biosafety Committees at the Connecticut Agricultural Experiment Station and Iowa State University.

Note that full information on the approval of the study protocol must also be provided in the manuscript.

Plants

Seed stocks	N/A
Novel plant genotypes	N/A
Authentication	N/A
